# Retrospective cohort study measuring the disparities in post-COVID conditions among persons with disabilities: findings from primary care settings, 2019–22

**DOI:** 10.1093/rescon/vmag125

**Published:** 2026-07-14

**Authors:** Esther E. Velasquez, Robert L. Phillips, JoAnn M. Thierry, Sharon Saydah, Emilia H. Koumans, Ilia Rochlin, Linda Geng, David Rehkopf, Neil Kamdar

**Affiliations:** 1 Center for Population Health Sciences, Stanford School of Medicine, Stanford University, Palo Alto, CA 94306, United States; 2 American Board of Family Medicine, Washington, DC 20036, United States; 3 National Center on Birth Defects and Developmental Disabilities, Centers for Disease Control and Prevention, Atlanta, GA 30333, United States; 4 National Center for Immunization and Respiratory Diseases, Centers for Disease Control and Prevention, Atlanta, GA 30333, United States; 5 Office of Public Health Data, Surveillance, and Technology, Centers for Disease Control and Prevention, Atlanta, GA 30333, United States

**Keywords:** COVID-19, disabilities, post-COVID conditions, primary healthcare, health disparities

## Abstract

**Background and aims:**

This study compared the incidence of post-COVID conditions by pre-pandemic disability status within primary care.

**Methods:**

This retrospective observational study used electronic health record data to identify primary care patients with ≥1 visit during January 2018–December 2019 and ≥1 visit during May 2020–December 2022. Disability status was identified using diagnostic codes from January 2018 to December 2019. COVID-19 diagnosis was captured during April 2020–December 2022. Accelerated Failure Time models were used to assess time to incident circulatory, endocrine, neurological, and respiratory diagnoses at least 28 days post COVID-19.

**Results:**

Among patients aged ≥50 years, those with disabilities and COVID-19 had higher incidence and earlier onset of newly diagnosed circulatory [time ratio (TR) 0.70, 95% confidence interval (CI) 0.64–0.77], endocrine (TR 0.91, 95% CI 0.84–0.99), and neurological (TR 0.52, 95% CI 0.44–0.62), but not respiratory conditions during the post-COVID period as compared to patients with COVID-19 without disability. No associations were found among patients aged <50 years.

**Conclusion:**

Primary care patients with pre-existing disability experienced a higher incidence and earlier onset of post-COVID conditions than patients without disability.

## Introduction

The COVID-19 pandemic differentially affected persons with disabilities due to existing underlying differences in health and social needs, which resulted in increased risk for persons with disabilities [1–3]. Persons with disabilities are more likely to be living in group quarters or institutional settings, to rely on mass transit for transportation, to live in poverty, and to have less than a high school diploma [1]. The pandemic disrupted access to work, to caregivers, and to routine healthcare and rehabilitation services; persons with disabilities had reduced access to appropriate personal protective equipment but increased exposure to others [3]. Compared to persons with no disabilities, persons with disabilities were more likely to be hospitalized for COVID-19, and among those hospitalized, persons with disabilities had increased risk for mechanical ventilation, severe COVID-19, readmission, longer hospital stay, in-hospital mortality, and 30-day mortality [4–7]. Most of our understanding of the health consequences of COVID-19 for persons with disabilities comes from inpatient data or a composite of inpatient and outpatient reports. Additionally, information is lacking on the incidence of post-COVID conditions (PCCs) for persons with disabilities who were treated in primary care settings.

Several proposed risk factors for PCC overlap with characteristics of persons with disabilities in the USA. These include older age, pre-existing comorbidities and/or psychiatric disorders, absence of a COVID-19 vaccination, number of symptoms and severity of acute COVID-19, and length of hospital stay for COVID-19 [8, 9]. Compared to adults without disabilities, persons with disabilities in the USA are more likely to be obese, smoke, and have diabetes and heart disease, among other clinical comorbidities [10–12]. Given the elevated prevalence of risk factors and overall clinical burden of disease, persons with disabilities may be more likely to experience PCC. In fact, self-reported data from the National Survey on Health and Disability suggests that the prevalence of PCC is 40.6% among individuals with pre-existing disabilities compared to 18.9% in the general population using data from the Household Pulse Survey [13]. Yet, research using clinical data, which includes clinician-documented diagnoses and longitudinal follow-up, is needed to corroborate and build upon self-reported findings.

We explored whether persons with disabilities and COVID-19 documentation in primary care are more likely to have a PCC-related diagnosis and an earlier PCC-related diagnosis compared to persons without disabilities and COVID-19. This study helps illuminate the current state of persons with disabilities who do and do not have COVID-19 and potential clinical consequences. We estimate the incidence of newly documented circulatory, endocrine, neurological, and respiratory diagnoses stratified by disability status and COVID-19 documented at primary care encounters.

## Methods

### Data source

We derived data for this retrospective observational study from the American Family Cohort (AFC), a dataset of electronic health records (EHRs) for over 8 million patients from clinics located in 47 US states. The AFC comprises clinics participating in the PRIME Registry, a national Qualified Clinical Data Registry for primary care established by the American Board of Family Medicine in 2016 [14]. The PRIME Registry contains information commonly found in EHRs including patient demographics, visit diagnosis codes, patient problem lists, procedures, and medications.

### Data availability statement

The AFC data used for this study are accessible to researchers; additional details as well as applications for access are available through the AFC Research Consortium (https://americanfamily-cohort.org).

### Patient consent statement

This protocol was reviewed and approved by the local Administrative Panels for the Protection of Human Subjects (#61956). Because all data were retrospective and investigators did not have access to or permission to contact patients, a waiver of informed consent was granted.

### Study population

The study population required two inclusion criteria: (1) patients must have had at least one primary care visit during 1 January 2018–31 December 2019 and (2) patients must have at least one primary care visit during 1 May 2020–30 December 2022 recorded in the PRIME Registry. We chose these criteria to restrict the analytic sample to patients who had established care at a practice prior to the COVID-19 pandemic, and to identify underlying health conditions for visits during the pre-pandemic period.

There were 3 142 123 AFC patients with one or more primary care visits during January 2018–December 2019. Of those patients, 2 662 873 also had at least one visit during 1 May 2020–30 December 2022. We excluded participants whose first congenital or acquired disability diagnosis was documented during a primary care visit after 31 December 2019 (*n* = 67 870), to ensure that the disabilities included in the analysis occurred prior to the COVID-19 pandemic. The final patient population was 2 595 003, and patients with ≥1 disability (*n* = 126 215) comprised 4.9% of the study population.

### Disability status

We identified disability status using *International Classification of Diseases, 10th Revision* (ICD-10-CM) diagnosis codes and grouped them by disability type: hearing, intellectual and developmental, mobility, vision, or other (Table S1). Diagnosis codes indicating disability were defined by subject-matter experts and informed by previously published literature [6] and the Centers for Medicare and Medicaid Services Chronic Condition Data Warehouse [15]. Patients were identified with a disability if they had one or more disability diagnosis codes in the EHR “problem list” or in the visit diagnosis during 1 January 2018–31 December 2019 and were assumed chronic throughout the study period. Since patients can frequently experience multiple disabilities, disability types defined for this study were not mutually exclusive [16]. Patients with no documented diagnosis codes for specific disabilities during the 2018–19 period were classified as the “No disability” category.

### COVID-19 definition

We classified patients as having primary care documented COVID-19 if they received an ICD-10-CM diagnosis code of U07.1 or U07.2 during a visit on or after 1 April 2020, until the end of the patient follow-up. Patients without COVID-19 diagnosis codes in their primary care EHR were classified as not having had primary care documented COVID-19.

### Covariates

Sex, race, ethnicity, and age were extracted from the EHR. Race and ethnicity categories were combined into a single variable to account for both race and Hispanic/Latino ethnicity. Underlying medical conditions clinically deemed to have an increased risk of severe COVID-19 were informed by a previously published list [17]. We assessed 19 underlying pre-existing medical conditions and identified them using ICD-10-CM diagnosis codes in the patient EHR during 1 January 2018–31 December 2019 (Table S2). Possible counts of underlying medical conditions for an individual patient could range from 0 to 19, and we grouped patients into three strata: none, one, or more than one underlying medical condition.

### Outcomes

We assessed four clinical outcomes: circulatory, endocrine, neurological, and respiratory diagnoses, which could signal PCCs documented in the primary care setting after the index COVID-19 diagnosis date (referred to as “incident”). All documented PCCs were obtained through diagnosis codes at the time of the visit or through the EHR problem list. We used previously published work based on parent ICD-10-CM codes for each clinical outcome group: circulatory (I00-I99), endocrine (E00-E89), neurological (neurological conditions parent codes: A85, A86, F01-F03, F05, G04, G05, G26, G31, G40, G41, G43, G44, G50-G59, G61-G65, R26, R27, R29, R40, R41, R44, R51), and respiratory (J00-J99). These outcomes encompass the most frequently reported PCC symptoms [7, 18]. To identify new primary care-diagnosed PCCs, we compared all diagnosis codes assigned at visits prior to the index date with those conditions assigned after the index date. For COVID-19 patients, the index date is the date in the EHR that the patient was first diagnosed with COVID-19 by their primary care provider. In accordance with the Centers for Disease Control and Prevention definition of PCCs, conditions must be diagnosed 28 days or more following COVID-19 diagnosis [19]. For example, if a patient received a circulatory diagnosis 12 days after their COVID-19 diagnosis date recorded in the EHR, we did not consider this a PCC. However, if this patient received a circulatory diagnosis on day 28 or later following their COVID-19 diagnosis, this was considered a PCC. For non-COVID-19 patients, we randomly assigned an index date on or between 1 April 2020 and their last date of follow-up without requiring a 28-day wash-out period following the index date. The purpose of assigning an index date to non-COVID-19 patients was to balance person-time across the two groups and limit systematic age bias if we fixed an earlier index date for the non-COVID-19 patient controls [20–23].

### Statistical analysis

We described differences in age, race/ethnicity, sex, and underlying medical conditions by disability and COVID-19 status. Next, we estimated the crude incidence rate of incident circulatory, endocrine, neurological, and respiratory diagnoses during the post-index period by disability status and COVID-19 status. The post-index period is bounded by the index date and the end of follow-up. The end of follow-up for patients who experience the outcome was the first date of diagnosis of the PCC outcome in the primary care setting. For patients who do not experience the outcome, the end of follow-up is the last visit date of any patient at the practice that the patient attends. These observable periods constitute diagnosis-free person-time for each patient.

To estimate the ratio of disease-free survival for new circulatory, endocrine, neurological, and respiratory conditions, we used parametric Accelerated Failure Time models (Table 2) [24]. Our first model (model 1) included main effects for COVID-19 and disability status with an interaction term. Using incremental risk adjustment, we estimated a second model (model 2) that appended covariates for age, sex, and race/ethnicity. Our final model (model 3) included the same covariates as model 2 and added adjustment for the number of underlying medical conditions. To quantify differential effects on the unadjusted and adjusted ratio of survival times, herein referenced as the time ratio (TR), we post-estimated the conditional TR such that between and within comparisons of COVID-19 and disability status could be obtained. To aid interpretation, we selected 100 calendar days as a reference length of time to quantify the total number of days longer or shorter to incident PCCs. Therefore, adjusted TRs that were >1 could be used to calculate the incremental number of days to the incident PCC compared to the reference category for the covariate. Conversely, for TRs <1, this would enable ease of interpretation to quantify how many fewer days to the incident PCC compared to the reference category for the covariate.

As a secondary analysis, we repeated the modeling process described above to estimate the TR of PCC disease-free survival and stratified by four disability types and then separately by three age groups (Tables S3–S5). Fifty years was chosen for the lower-bound threshold of the oldest age group since this is the age above which COVID-19 therapeutics are recommended for those without a pre-existing co-morbidity or disability [25, 26].

Data analysis was conducted using R version 4.2.2 (2022 The R Foundation for Statistical Computing Platform) and with Statistical Analysis System, version 9.4 (SAS Institute, Cary, NC).

## Results

Demographic characteristics and underlying medical conditions by disability and COVID-19 status are summarized in Table 1. First, we summarize PCCs among patients with disabilities, comparing those with COVID-19 to those without. Among patients with disabilities, patients with COVID-19 had an additional 446 new circulatory diagnoses, 572 new endocrine diagnoses, 123 new neurological diagnoses, and 701 new respiratory diagnoses per 100 000 person-months as compared to patients without COVID-19 (Fig. 1). Using 100 days as a reference, time to PCC-related diagnosis among patients with disability and COVID-19 was 51 days shorter for receiving a new circulatory diagnosis (TR 0.49, 95% CI 0.45–0.54), 48 days shorter for a new endocrine diagnosis (TR 0.52, 95% CI 0.48–0.56), 49 days shorter for a new neurological diagnosis (TR 0.51, 95% CI 0.44–0.60), and 61 days shorter for a new respiratory diagnosis (TR 0.39, 95% CI 0.36–0.41) as compared to their counterparts with disability and no COVID-19 (Table 2A).

Second, among patients with COVID-19, patients with disabilities had an additional 339 new circulatory diagnoses, 292 new endocrine diagnoses, 130 new neurological diagnoses, and 97 new respiratory diagnoses per 100 000 person-months as compared to patients without COVID-19 (Fig. 1).

Third, using 100 days as a reference, time to new diagnosis among patients with COVID-19 and disability was 20 days shorter for new circulatory diagnosis (TR 0.80, 95% CI 0.74–0.87), 8 days shorter for new endocrine diagnosis (TR 0.92, 95% CI 0.86–0.99), and 40 days shorter for new neurological conditions (TR 0.60, 95% 0.51–0.7) as compared to patients with COVID-19 and no disability (Table 2B). There was no difference in time to a new respiratory diagnosis. Fourth, we summarize PCCs among patients with both COVID-19 and disability, comparing them to patients with no COVID-19 and no disability. Compared to patients with no COVID-19 and no disability, patients with COVID-19 and disability had an additional 617 new circulatory diagnoses, 735 new endocrine diagnoses, 173 new neurological diagnoses, and 782 new respiratory diagnoses per 100 000 person-months (Table 2). Using 100 days as a reference, time to new diagnosis among patients with COVID-19 and disability was 61 days shorter for new circulatory (TR 0.39, 95% CI 0.36–0.42), 53 days shorter for new endocrine (TR 0.47, 95% CI 0.44–0.50), 71 days shorter for new neurological (TR 0.29, 95% CI 0.25–0.34), and 65 days shorter for respiratory diagnoses (TR 0.35, 95% CI 0.33–0.37) compared to patients with no COVID-19 and no disability (Table 2C).

## Discussion

We found that COVID-19 is associated with an increased risk and earlier diagnosis of PCCs among persons with disabilities in primary care settings. There was an increase in crude incidence of newly diagnosed circulatory, endocrine, neurological, and respiratory diagnoses after index COVID-19 after adjustment. Furthermore, persons with disabilities and COVID-19 experienced circulatory, endocrine, and neurological conditions sooner compared to those without disability and COVID-19. Noteworthy, however, was that there were no differences in respiratory condition incidence.

Existing research suggests that adults with pre-existing disabilities experienced an increased risk of hospitalization, invasive mechanical ventilation, and death compared to individuals without disability [4, 6]. And since severe COVID-19 is a risk factor for PCCs [8, 9], the higher incidence of PCCs might be expected among persons with disabilities after COVID-19. Yet, this does not sufficiently account for our findings since the cohort consists of patients treated for COVID-19 in primary care. Thus, our findings expand current evidence supporting that patients with disabilities have an increased risk of PCCs in the event of a more moderate presentation of COVID-19.

The secondary analyses were motivated by existing evidence that COVID-19 outcomes during the first 28 days following initial illness vary by demographic characteristics and underlying health status. Since the findings from these studies are mixed, it is difficult to disentangle the interplay of demographic characteristics, co-morbidities, and disability [4–7, 27, 28]. In some cases, the association of disability and adverse COVID-19 outcomes is eliminated by the inclusion of covariates for adjustment. For example, among 1279 patients hospitalized for COVID-19, patients with disabilities had 1.77 times the rate of 30-day readmission (95% CI 1.14–2.75) and longer hospital stay [5]. Yet, the risk of in-hospital death as well as ICU admission was no longer statistically significant after adjustment for age, comorbidities, sex, dementia or psychiatric disorders, and residence type.

There is also evidence that the association between COVID-19 and adverse outcomes persists for only some subgroups after adjustment. In a US study of 6500 hospitalized patients <65 years with disabilities (defined as patients or proxies who reported assistive needs due to vision, hearing, cognitive, developmental, physical/mobility, or respiratory difficulties) had 1.35 times (95% CI 1.01–1.81) the hazard of developing severe COVID-19 during hospitalization, but not among patients ≥65 years [29]. Similarly, in a US cohort identified using inpatient and outpatient EHR data from 42 healthcare organizations, patients with developmental disabilities had a greater 30-day case fatality rate, a finding which persisted across all age groups but was most apparent in those aged ≤17 years [7]. These findings suggest that there may be increased disparities in severe COVID-19 outcomes by disability status for younger persons. In our analyses stratified by age, we did not find differences in PCCs by disability status among individuals aged <50 years. Research using self-report data from the National Health Interview Survey found that the odds of PCC were 2 times greater for individuals whose disability started before age 22 [30]. Further research that accounts for both age at COVID-19 infection and age at disability is needed to better understand differential age effects.

We recognize the following limitations of this analysis. The generalizability is limited to those individuals seeking care. Plausibly, patients with disability without a usual primary care practice may be substantively different in their presentation and incidence of PCCs. Further, disability, COVID-19, and PCC were dependent upon having a documented diagnosis; thus, misclassification is possible. Specifically, laboratory data were not used for confirmation of COVID-19 diagnosis. We were unable to account for the severity of disability or COVID-19 presentation, and certain treatments for COVID-19 were not captured, which could potentially account for differences in PCC incidence by disability and COVID-19 status. While vaccination data are available in the dataset, it was not included in the analysis as previous work has demonstrated that while more than 90% of primary care clinics had routine vaccination data, more than 40% had no record of COVID-19 vaccination receipt or status [31]. Thus, COVID-19 vaccination status was not reliable across the entire dataset at the time of the study and reflects a limitation of the study. Additionally, the EHR did not include an exit date for patients, and we may have overestimated follow-up periods for patients who were no longer attending the same healthcare setting. Finally, we did not assess the diagnosis of post COVID-19 (U09.9), but rather PCC-related diagnoses, and thus, these findings may not be generalizable to comparisons made using the U09.9 diagnosis.

## Conclusion

The current work expands our understanding of the impacts of COVID-19 on the health of individuals with disabilities. The volume of patients seeking care in the primary care setting is nearly 15 times higher than those receiving care in hospitals [32]; therefore, using real-world primary care data to address this question is important for guiding care and patient and caregiver concerns.

The magnitude of our findings for circulatory and neurological outcomes underscores the importance of primary care clinical protocols for people with disabilities or caregivers who support them after a COVID-19 diagnosis. While our findings are retrospective, they demonstrate the value of large, outpatient clinical registries for detecting differential risk and effects during an epidemic. Their routine use during an epidemic could offer early options to help clinical practices adjust patient population monitoring and treatment in a timely way.

## Supplementary Material

supplementary 2

supplementary 1

Supplementary material is available at *Research Connections* online.

## Figures and Tables

**Figure 1:**
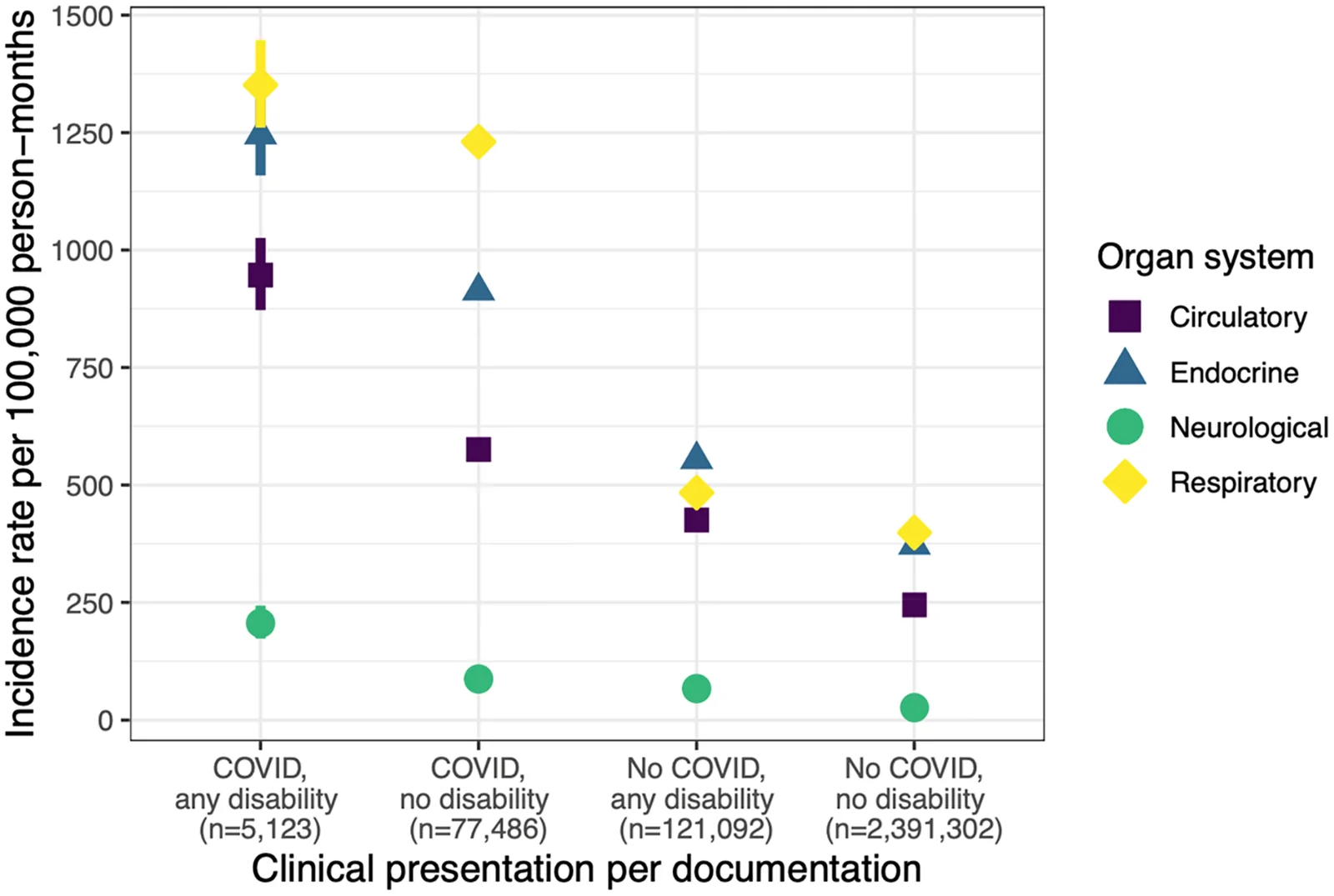
Crude incidence of post-COVID conditions as documented in the primary care setting per 100 000 person-months of observation by disability and COVID-19 status. Horizontal bars indicate 95% confidence intervals. Confidence intervals are too small to be visible on three of the clinical outcomes due to large sample sizes. Patients were from the American Family Cohort and (1) must have had at least one primary care visit during 1 January 2018–31 December 2019 and (2) at least one primary care visit on or after 1 May 2020. Patients may have had more than one of the outcomes. ICD-10-CM codes for the outcomes are as follows: circulatory (I00-I99), endocrine (E00-E89), respiratory (J00-J99), and neurological (A85, A86, F01-F03, F05, G04, G05, G26, G31, G40, G41, G43, G44, G50-G59, G61-G65, R26, R27, R29, R40, R41, R44, R51)

**Table 1:** Demographic characteristics and underlying medical conditions by disability and COVID-19 status, January 2018–December 2022.

	Pre-existing disability^a^		No pre-existing disability^a^	
	No COVID-19 *N* (%) (*N* = 121 092)	COVID-19^b^ *N* (%) (*N* = 5123)	No COVID-19 *N* (%) (*N* = 2 391 302)	COVID-19^b^ *N* (%) (*N* = 77 486)
Age (years) at last visit				
0–19	20 236 (16.7)	429 (8.4)	329 222 (13.8)	5595 (7.2)
20–34	8854 (7.3)	297 (5.8)	381 360 (15.9)	8004 (10.3)
35–44	6821 (5.6)	323 (6.3)	284 846 (11.9)	9619 (12.4)
45–54	10 354 (8.6)	546 (10.7)	325 391 (13.6)	13 322 (17.2)
55–64	16 246 (13.4)	781 (15.2)	396 036 (16.6)	16 527 (21.3)
65–74	22 304 (18.4)	1084 (21.2)	358 648 (15.0)	14 018 (18.1)
75+	35 799 (29.6)	1653 (32.3)	295 969 (12.4)	10 149 (13.1)
Missing	478 (0.4)	10 (0.2)	19 830 (0.8)	252 (0.3)
Sex				
Female	64 877 (53.6)	2928 (57.2)	1 326 134 (55.5)	44 298 (57.2)
Male	55 697 (46.0)	2185 (42.7)	1 044 665 (43.7)	32 933 (42.5)
Unknown	518 (0.4)	10 (0.2)	20,503 (0.9)	255 (0.3)
Race/ethnicity				
American Indian or Alaska Native	507 (0.4)	28 (0.5)	10,805 (0.5)	393 (0.5)
Asian or Pacific Islander	2643 (2.2)	69 (1.3)	55 656 (2.3)	1211 (1.6)
Black or African American	10 363 (8.6)	342 (6.7)	171 740 (7.2)	4964 (6.4)
Hispanic or Latino	13 069 (10.8)	736 (14.4)	227 603 (9.5)	8459 (10.9)
White	77 742 (64.2)	3579 (69.9)	1 536 527 (64.3)	56 213 (72.5)
Unknown	16 768 (13.8)	369 (7.2)	388,971 (16.3)	6,246 (8.1)
Underlying medical conditions^c^				
None	46 380 (38.3)	1387 (27.1)	1 520 206 (63.6)	39 698 (51.2)
One	30 861 (25.5)	1373 (26.8)	509 962 (21.3)	20 478 (26.4)
Two or more	43 851 (36.2)	2363 (46.1)	361 134 (15.1)	17 310 (22.3)

a Pre-existing disability was based on diagnosis codes in the problem list for the patient or the primary care visit diagnosis codes for any visit from 1 January 2018 to 31 December 2019.

b COVID-19 status was defined as patients with documented diagnosis codes U07.1 or U07.2 during a visit on or after 1 April 2020 until the end of the follow-up period (30 December 2022).

c We assessed 19 different categories of underlying pre-existing medical conditions and identified them using ICD-10-CM diagnosis codes in the patient EHR during 1 January 2018–31 December 2019.

**Table 2:** Parametric AFT models and adjusted ratios of days to outcome for those with and without COVID-19 and/or disability.

				Time ratio			Estimated days earlier to outcome (reference 100 days)		
	Exposed	Reference	Outcome	TR	LB CI	UB CI	Days	LB CI	UB CI
A	COVID-19 + disability	No COVID-19 + disability	Circulatory	0.49	0.45	0.54	51	55	46
			Endocrine	0.52	0.48	0.56	48	52	44
			Neurological	0.51	0.44	0.60	49	56	40
			Respiratory	0.39	0.36	0.41	61	64	59
B	COVID-19 + disability	COVID-19 + no disability	Circulatory	0.80	0.74	0.87	20	26	13
			Endocrine	0.92	0.86	0.99	8	14	1
			Neurological	0.60	0.51	0.70	40	49	30
			Respiratory	0.99	0.92	1.06	1	8	−6
C	COVID-19 + disability	No COVID-19 + no disability	Circulatory	0.39	0.36	0.42	61	64	58
			Endocrine	0.47	0.44	0.50	53	56	50
			Neurological	0.29	0.25	0.34	71	75	66
			Respiratory	0.35	0.33	0.37	65	67	63

LB CI and UB CI: lower and upper bounds of the 95% confidence intervals of the ratio of the survival times; TR: ratio of the event times between the exposed and reference groups. The statistical models presented include: COVID-19 + disability + COVID-19 × disability + age + sex + race/ethnicity + underlying medical conditions.

## Data Availability

The AFC data used for this study are accessible to researchers; additional details as well as applications for access are available through the American Family Cohort (AFC) Research Consortium (https://americanfamily-cohort.org).
