# Supplementary material for: Retrospective cohort study measuring the disparities in post-COVID conditions among persons with disabilities: findings from primary care settings, 2019–22
Source: Res Connect. Author manuscript; Available in PMC 2026 Oct 24. (PMC13693701; doi:10.1093/rescon/vmag125)
Supplement: Supplementary file 1 [file NIHMS2212073-supplement-supplementary_2.docx]

**Supplemental Methods**

We chose the Accelerated Failure Time modeling approach due to different patient follow-up times and to estimate ratios of times to incident PCC between groups with and without adjustment.^22,23^ Patients were right censored if they did not experience the clinical outcome during follow-up. We assessed different candidate distributions (Weibull, log-logistic, Gaussian, and Gamma) and model fit statistics minimizing log likelihood and the Akaike Information Criterion (AIC) for each model. After consideration of different candidate distributions, we applied a Weibull distribution to all models. Since we hypothesized that there would exist differential effects in disease-free survival time conditional on COVID-19 and disability status, we applied three principal models with an interaction effect. Our first model (Model 1) included main effects for COVID-19 and disability status with an interaction term. We also postulated that the main and joint effects of COVID-19 and disability status may be attenuated by adjustment for other observable characteristics, so we implemented additional models with covariates via block inclusion. Using incremental risk adjustment, we estimated a second model (Model 2) that appended covariates for age, sex, and race/ethnicity. Our final model (Model 3) included the same covariates as Model 2 and added adjustment for underlying medical conditions. To quantify differential effects on the unadjusted and adjusted ratio of survival times, herein referenced as the time ratio (TR), we post-estimated the conditional time ratio such that between and within comparisons of COVID-19 and disability status could be obtained.

***Sensitivity Analysis Methods***

Since the AFC is an open real-world cohort and not a controlled study with required study visits, and persons with disabilities may be more likely to actively use health care, we performed additional statistical tests to determine whether our findings are driven by differences in health care utilization. Specifically, as a robustness check of the main findings, we assessed whether healthcare utilization or differences in the distribution of the index dates (which also could be influenced by healthcare utilization) between patients with COVID-19 documented at a primary care encounter and patients without documented COVID-19 could explain differences in presence and timing of primary care documented PCC between patient groups. Healthcare utilization was observed from January 1, 2018 to December 31, 2019 defined as a categorical variable: 1-2 visits, 3-5 visits, and 6 or more visits. First, as an alternative method of assigning index dates to patients without COVID-19, we randomly sampled from all observed index dates for patients with COVID-19 at the clinic the patient attended. Thus, each patient control had an index date in which a patient at the same clinic was diagnosed with COVID-19. This resulted in a more parallel distribution of index dates between patients with and without COVID-19. Second, we also adjusted for healthcare utilization.

***Sensitivity Analysis Results:*** Results of the sensitivity analysis adjusting for pre-pandemic healthcare utilization and assigning index dates to patients without COVID-19 based on observed index dates for patients with COVID-19 overall did not show substantial differences with our primary findings (Table S7). The most notable differences in point estimates were comparing persons with disabilities to persons without disabilities among patients with documented COVID-19, where we observed time ratios closer to the null for endocrine and neurological outcomes in the sensitivity analyses. The time ratio for endocrine related outcomes changed from 0.92 (95% CI 0.86-0.99) to 0.98 (95% CI 0.90, 1.06), and for neurological related outcomes from 0.60 (95% CI 0.51-0.70) to 0.75 (95% CI 0.63-0.88). In all cases, 95% confidence intervals of the time ratio estimates were overlapping between our main analysis and the sensitivity analysis.

**Supplemental Analysis**

Supplemental analyses of the top five diagnoses made in the primary care setting are reported, stratified by persons with and without disabilities, whether they had a documented COVID diagnosis, and specifically the clinical outcome group (e.g., circulatory, endocrine, neurological, or respiratory) (Table S6). Amongst persons with disabilities, the most frequently diagnosed circulatory conditions included essential (primary) hypertension, atherosclerotic heart disease, hypotension, hypertensive chronic kidney disease, and heart failure. The most frequently diagnosed endocrine conditions were mixed and/or unspecified hyperlipidemia, Vitamin D deficiency, and Type 2 diabetes. The most frequently diagnosed neurological conditions included altered mental status, unsteadiness on feet, repeated falls, polyneuropathy, and unspecified dementia. Finally, the most frequently diagnosed respiratory conditions included acute upper respiratory infection, acute sinusitis, acute bronchitis, acute pharyngitis, and pneumonia.

**Table S1. ICD-10-CM Codes Used to Identify Individuals with Disability by Disability Type**

| **Disability Type** | **ICD-10-CM Code** |
| --- | --- |
| Hearing | H90.1*, H90.3, H90.4*, H90.5, H90.6, H90.7*, H90.8, H90.A2*, H90.A3*, H91.0*, H91.3, H91.8*, H91.9*, S04.6* |
| Intellectual | D81.810, E00*, E03.1, E03.2, E70.0, E70.2*, E71.0, E71.11*, E71.19, E71.2, E72.11, E72.2*, E72.3, E72.4, E72.5, E72.50, E72.52, E72.59, E72.8*, E74.2*, E74.8*, E78.71, E78.72, E79.1, F70, F71, F72, F73, F78, F79, F80*, F82, F84*, F88, F89, G31.84, H93.25, P04.3, Q00*, Q01*, Q02, Q03*, Q04*, Q85.1, Q86.0, Q87.1*, Q87.2, Q87.3, Q87.5, Q87.8*, Q89.7, Q89.8, Q90*, Q91*, Q92*, Q93*, Q95.2, Q95.3, Q99.2, R48.0 |
| Mobility | E70.330, E70.5, G04.1, G11.4, G35, G36*, G37.1, G37.2, G37.3, G37.4, G37.8, G37.9, G71.0*, G71.11, G71.2*, G80*, G81*, G82*, G83*, G90.1, I69.03*, I69.04*, I69.05*, I69.06*, I69.13*, I69.14*, I69.15*, I69.16*, I69.23*, I69.24*, I69.25*, I69.26*, I69.33*, I69.34*, I69.35*, I69.36*, I69.83*, I69.84*, I69.85*, I69.86*, I69.93*, I69.94*, I69.95*, I69.96*, Q05*,Q06*, Q07*, R26*, S14*, S24*, S34*, Z74.09 |
| Visual | E70.3*, H53.0*, H53.10, H53.11, H53.13*, H53.16, H53.19, H53.2, H53.3, H53.30, H53.32, H53.34, H53.4*, H53.5*, H53.6*, H53.8, H53.9, H54, Q04.4, S04.0* |
| Other | E70.8, S06.1, S06.2, S06.3, S06.4, S06.5, S06.6, S06.8, S06.9, Z73.6, Z73.82 |

*An asterisk indicates that the parent code (e.g. H90.1*) would include all IDC-10-CM codes that have H90.1 including any character for the final position. Therefore, parent codes reflect the prefix of either length 3 or 4 characters in accordance with the ICD-10-CM diagnosis codeset, and asterisks are wildcard characters.

**Table S2. ICD-10 Codes used to Identify Underlying Medical Conditions**

| Condition | ICD-10-CM Code |
| --- | --- |
| Asthma | J45.4*, J45.5*, J45.9*, J45.99* |
| COPD | J40*, J41*, J42*, J43*, J44* |
| Cystic fibrosis | E84* |
| Pulmonary fibrosis | J84* |
| Other lung conditions | A15*, A31.0, B39*, B40*, B41*, B44*, B45*, B46.0, D86.0, E88.01, J47*, J60*, J61*, J62*, J63*, J64*, J65*, J66*, J67*, J68*, J69*, J70*, J81*, J82*, J95*, J96*, J98.1*, J99*, P25*, P26*, P27*, P28*, Q33*, T86.3*, T86.8*, Z94.2 |
| Heart disease | I01*, I02*, I05*, I06*, I07*, I08*, I09*, I11*, I13*, I21*, I22*, I23*, I24*, I25*, I26*, I27*, I28*, I31*, I34*, I35*, I36*, I37*, I41*, I42*, I43*, I44*, I46*, I48*, I50*, I51*, I52*, I71*, I72*, I73, I74*, I75*, I79*, I97.0, I97.1*, M31*, Q20*, Q21*, Q22*, Q23*, Q24*, Q25*, Q26*, Q27.0, Q27.3*, Q27.4, Q27.8, Q27.9, Q28*, Q89.3, Z94.1, Z95*, Z98.61 |
| Hypertension | I10* |
| Sickle cell and thalassemia | D56.0, D56.1, D56.2, D56.5, D56.9, D57.0, D57.1, D57.2, D57.4, D57.8 |
| Cancer | C00*, C01*, C02*, C03*, C04*, C05*, C06*, C07*, C08*, C09*, C10*, C11*, C12*, C13*, C14*, C15*, C16*, C17*, C18*, C19*, C20*, C21*, C22*, C23*, C24*, C25*, C26*, C30*, C31*, C32*, C33*, C34*, C37*, C38*, C39*, C40*, C41*, C43*, C44*, C45*, C46*, C47*, C48*, C49*, C4A*, C50*, C51*, C52*, C53*, C54*, C55*, C56*, C57*, C58*, C60*, C61*, C62*, C63*, C64*, C65*, C66*, C67*, C68*, C69*, C70*, C71*, C72*, C73*, C74*, C75*, C76*, C77*, C78*, C79*, C7A*, C7B*, C80*, C81*, C82*, C83*, C84*, C85*, C86*, C88*, C90*, C91*, C92*, C93*, C94*, C95*, C96*, D03*, D46* |
| Cerebrovascular diseases | I60*, I61*, I62*, I63*, I67*, I68*, I69* |
| Neurological/Musculoskeletal | E75.02, E75.19, E75.4, F01*, F02*, F03*, F71*, F72*, F73*, F84.2, G10*, G11*, G12*, G13*, G14*, G20*, G21*, G23*, G24*, G25*, G26*, G30*, G31*, G32*, G35*, G36*, G37*, G40*, G45*, G46*, G60*, G61*, G62*, G63*, G64*, G70*, G71*, G73*, G80*, G81*, G82*, G83*, G90.3, G91*, G93*, G94*, G95*, G99.2, H49.81, M12.0, M36.0, P91*, Q00*, Q01*, Q02*, Q03*, Q04*, Q05*, Q06*, Q07*, Q76*, Q77*, Q78*, Q79*, Q85*, Q87.4, Q91*, Q92*, Q93*, Q96*, R41*, R53.2, R54* |
| Diabetes | E08*, E10*, E11*, E13* |
| Overweight | E66.3, Z68.25, Z68.26, Z68.27, Z68.28, Z68.29 |
| Obesity | E66.09, E66.1, E66.8, Z68.3, E66.9 |
| Severe obesity | E66.01, E66.2, Z68.4 |
| Liver disease | B18*, I81*, I85*, K70*, K71*, K72*, K73*, K74*, K75*, K76*, K77* |
| Chronic kidney disease including dialysis | I12*, N18*, Z49*, Z94.0*, Z99.2*, N01*, N02*, N03*, N04*, N05*, N06*, N07*, N08*, N11*, N14*, N15*, N16*, N25*, N26*, N28*, Q60*, Z91.15, Q27.1, Q27.2 |
| Immunosuppression | B20*, B59*, B97.3, D47.Z1, D70*, D71*, D72*, D73*, D76*, D80*, D81*, D82*, D83*, D84*, D89*, M05*, M06*, M07*, M08*, M30*, M31*, M32*, M33*, M34*, M35.0, M35.9, Q89.0, Z21*, Z48.2, Z51.0, Z51.1, Z79.5, Z94*, T86* |
| Substance use disorder | F11.10, F11.120, F11.121, F11.122, F11.129, F11.14, F11.150, F11.151, F11.159, F11.181, F11.182, F11.188, F11.19, F11.20, F11.21, F11.220, F11.221, F11.222, F11.229, F11.23, F11.24, F11.250, F11.251, F11.259, F11.281, F11.282, F11.288, F11.29, F11.90, F11.920, F11.921, F11.922, F11.929, F11.93, F11.94, F11.950, F11.951, F11.959, F11.981, F11.982, F11.988, F11.99, F14.10, F14.120, F14.121, F14.122, F14.129, F14.14, F14.150, F14.151, F14.159, F14.180, F14.181, F14.182, F14.188, F14.19, F14.20, F14.21, F14.220, F14.221, F14.222, F14.229, F14.23, F14.24, F14.250, F14.251, F14.259, F14.280, F14.281, F14.282, F14.288, F14.29, F14.90, F14.920, F14.921, F14.922, F14.929, F14.93, F14.94, F14.950, F14.951, F14.959, F14.980, F14.981, F14.982, F14.988, F14.99, F15.10, F15.120, F15.121, F15.122, F15.129, F15.14, F15.150, F15.151, F15.159, F15.180, F15.181, F15.182, F15.188, F15.19, F15.20, F15.21, F15.220, F15.221, F15.222, F15.229, F15.23, F15.24, F15.250, F15.251, F15.259, F15.280, F15.281, F15.282, F15.288 F15.29, F15.90, F15.920, F15.921, F15.922, F15.929, F15.93, F15.94, F15.950, F15.951, F15.959, F15.980, F15.981, F15.982, F15.988, F15.99, F12.10, F12.120, F12.121, F12.122, F12.129, F12.150, F12.151, F12.159, F12.180, F12.188, F12.19, F12.20, F12.21, F12.220, F12.221, F12.222, F12.229, F12.23, F12.250, F12.251, F12.259, F12.280, F12.288, F12.29, F12.90, F12.920, F12.921, F12.922, F12.929, F12.93, F12.950, F12.951, F12.959, F12.980, F12.988, F12.99, F10.10, F10.120, F10.121, F10.129, F10.14, F10.150, F10.151, F10.159, F10.180, F10.181, F10.182, F10.188, F10.19, F10.20, F10.20, F10.21, F10.220, F10.221, F10.229, F10.230, F10.231, F10.232, F10.239, F10.24, F10.250, F10.251, F10.259, F10.26, F10.27, F10.280, F10.281, F10.282, F10.288, F10.29, F10.920, F10.921, F10.929, F10.93, F10.94, F10.950, F10.951, F10.959, F10.96, F10.97, F10.980, F10.981, F10.982, F10.988, F10.99, F13.10, F13.120, F13.121, F13.129, F13.14, F13.150, F13.151, F13.159, F13.180, F13.181, F13.182, F13.188, F13.19, F13.20, F13.21, F13.220, F13.221, F13.222, F13.229, F13.230, F13.231, F13.232, F13.239, F13.24, F13.250, F13.251, F13.259, F13.26, F13.27, F13.280, F13.281, F13.282, F13.288, F13.29, F13.90, F13.920, F13.921, F13.929, F13.930, F13.931, F13.932, F13.939, F13.94, F13.950, F13.951, F13.959, F13.96, F13.97, F13.980, F13.981, F13.982, F13.988, F13.99, F16.10, F16.120, F16.121, F16.122, F16.129, F16.14, F16.150, F16.151, F16.159, F16.180, F16.183, F16.188, F16.19, F16.20, F16.21, F16.220, F16.221, F16.229, F16.24, F16.250, F16.251, F16.259, F16.280, F16.283, F16.288, F16.29, F16.90, F16.920, F16.921, F16.929, F16.94, F16.950, F16.951, F16.959, F16.980, F16.983, F16.988, F16.99, F18.10, F18.120, F18.121, F18.129, F18.14, F18.150, F18.151, F18.159, F18.17, F18.180, F18.188, F18.19, F18.20, F18.21, F18.220, F18.221, F18.229, F18.24, F18.250, F18.251, F18.259, F18.27, F18.280, F18.288, F18.29, F18.90, F18.920, F18.921, F18.929, F18.94, F18.950, F18.951, F18.959, F18.97, F18.980, F18.988, F18.99, F19.10, F19.120, F19.121, F19.122, F19.129, F19.14, F19.150, F19.151, F19.159, F19.16, F19.17, F19.180, F19.181, F19.182, F19.188, F19.19, F19.20, F19.21, F19.220, F19.221, F18.222, F19.229, F19.23, F19.24, F19.250, F19.251, F19.259, F19.26, F19.27, F19.280, F19.281, F19.282, F19.288, F19.29, F19.90, F19.920, F19.921, F19.922, F19.929, F19.93, F19.94, F19.950, F19.951, F19.959, F19.96, F19.97, F19.980, F19.981, F19.982, F19.988, F19.99 |

*An asterisk indicates that the parent code (e.g. H90.1*) would include all IDC-10-CM codes that have H90.1 including any character for the final position. Therefore, parent codes reflect the prefix of either length 3 or 4 characters in accordance with the ICD-10-CM diagnosis codeset, and asterisks are wildcard characters.

**Table S3. Demographic Characteristics and Underlying Medical Conditions by Disability Type: American Family Cohort, Patients with a Disability Diagnosis January 1, 2018 to December 31, 2019**

|  | Hearing  n=34,489 | Intellectual  n=33,438 | Mobility  n=39,747 | Visual  n=30,032 | Other  n=1,461 |
| --- | --- | --- | --- | --- | --- |
| Age |  |  |  |  |  |
| 0-19yr | 2,729 (7.9) | 12,983 (38.8) | 2,062 (5.2) | 4,790 (15.4) | 69 (4.7) |
| 20-34yr | 1,781 (5.2) | 3,263 (9.8) | 1,804 (4.5) | 2,893 (9.3) | 113 (7.7) |
| 35-44yr | 1,540 (4.5) | 1,685 (5.0) | 2,012 (5.1) | 2,366 (7.6) | 98 (6.7) |
| 45-54yr | 2,575 (7.5) | 2,037 (6.1) | 3,212 (8.1) | 3,834 (12.4) | 99 (6.8) |
| 55-64yr | 4,702 (13.6) | 2,917 (8.7) | 5,635 (14.2) | 4,982 (16.1) | 246 (16.8) |
| 65-74yr | 7,991 (23.2) | 3,813 (11.4) | 8,195 (20.6) | 5,656 (18.2) | 273 (18.7) |
| 75+yr | 13,026 (37.8) | 6,620 (19.8) | 16,683 (42) | 6,407 (20.6) | 557 (38.1) |
| Missing age | 145 (0.4) | 120 (0.4) | 144 (0.4) | 104 (0.3) | 6 (0.4) |
| Gender |  |  |  |  |  |
| Woman/Girl | 18,363 (53.2) | 15,505 (46.4) | 22,959 (57.8) | 17,706 (57.1) | 709 (48.5) |
| Man/Boy | 15,972 (46.3) | 17,808 (53.3) | 16,625 (41.8) | 13,213 (42.6) | 745 (51.0) |
| Other/Unknown | 154 (0.4) | 125 (0.4) | 163 (0.4) | 113 (0.4) | 7 (0.5) |
| Race/Ethnicity |  |  |  |  |  |
| American Indian or  Alaska Native | 137 (0.4) | 174 (0.5) | 165 (0.4) | 108 (0.3) | 14 (1.0) |
| Asian or Pacific  Islander | 952 (2.8) | 514 (1.5) | 578 (1.5) | 922 (3.0) | 20 (1.4) |
| Black or African  American | 1,787 (5.2) | 2,928 (8.8) | 3,866 (9.7) | 3,171 (10.2) | 108 (7.4) |
| Hispanic or Latino | 3,555 (10.3) | 4,217 (12.6) | 3,118 (7.8) | 4,629 (14.9) | 95 (6.5) |
| Unknown | 4,546 (13.2) | 4,618 (13.8) | 5,264 (13.2) | 4,033 (13) | 209 (14.3) |
| White | 23,512 (68.2) | 20,987 (62.8) | 26,756 (67.3) | 18,169 (58.5) | 1,015 (69.5) |
| Underlying Conditions |  |  |  |  |  |
| None | 12,943 (37.5) | 17,834 (53.3) | 7,077 (17.8) | 13,132 (42.3) | 387 (26.5) |
| One | 9,211 (26.7) | 7,246 (21.7) | 10,797 (27.2) | 8,084 (26.1) | 345 (23.6) |
| More than one | 12,335 (35.8) | 8,358 (25.0) | 21,873 (55) | 9,816 (31.6) | 729 (49.9) |

NOTE: Categories are not mutually exclusive. There are a total of 126,215 unique patients with a disability.

**Table S4. Parametric Accelerated Failure Time (AFT) Models and Unadjusted and Adjusted Ratios of Days to Outcome for those with COVID-19 and Disability Compared to Referents and Stratified by Disability Type**

|  | **Referent** | |  | **Any disability** | | | **Hearing** | | | **IDD** | | | **Mobility** | | | **Visual** | | |
| --- | --- | --- | --- | --- | --- | --- | --- | --- | --- | --- | --- | --- | --- | --- | --- | --- | --- | --- |
|  | **COVID** | **Disability** | **Outcome** | **TR** | **LB** | **TR** | **TR** | **LB** | **UB** | **TR** | **LB** | **UB** | **TR** | **LB** | **UB** | **TR** | **LB** | **UB** |
| A. | No | Disability | Circulatory | 0.49 | 0.54 | 0.45 | 0.53 | 0.62 | 0.46 | 0.51 | 0.57 | 0.45 | 0.53 | 0.63 | 0.45 | 0.47 | 0.57 | 0.39 |
|  |  |  | Endocrine | 0.52 | 0.56 | 0.48 | 0.62 | 0.71 | 0.54 | 0.43 | 0.48 | 0.38 | 0.57 | 0.66 | 0.49 | 0.58 | 0.69 | 0.49 |
|  |  |  | Neurological | 0.51 | 0.60 | 0.44 | 0.62 | 0.86 | 0.45 | 0.51 | 0.63 | 0.42 | 0.47 | 0.62 | 0.35 | 0.62 | 0.88 | 0.43 |
|  |  |  | Respiratory | 0.39 | 0.41 | 0.36 | 0.49 | 0.57 | 0.43 | 0.36 | 0.41 | 0.32 | 0.36 | 0.41 | 0.32 | 0.38 | 0.43 | 0.33 |
|  |  |  |  |  |  |  |  |  |  |  |  |  |  |  |  |  |  |  |
| B. | No | No disability | Circulatory | 0.39 | 0.42 | 0.36 | 0.39 | 0.45 | 0.34 | 0.38 | 0.42 | 0.33 | 0.38 | 0.44 | 0.32 | 0.39 | 0.47 | 0.33 |
|  |  |  | Endocrine | 0.47 | 0.50 | 0.44 | 0.50 | 0.57 | 0.44 | 0.43 | 0.47 | 0.39 | 0.46 | 0.52 | 0.40 | 0.48 | 0.57 | 0.41 |
|  |  |  | Neurological | 0.29 | 0.34 | 0.25 | 0.34 | 0.47 | 0.25 | 0.24 | 0.29 | 0.20 | 0.22 | 0.29 | 0.17 | 0.29 | 0.41 | 0.21 |
|  |  |  | Respiratory | 0.35 | 0.37 | 0.33 | 0.40 | 0.46 | 0.35 | 0.37 | 0.41 | 0.33 | 0.30 | 0.34 | 0.26 | 0.31 | 0.36 | 0.28 |
|  |  |  |  |  |  |  |  |  |  |  |  |  |  |  |  |  |  |  |
| C. | Yes | No disability | Circulatory | 0.80 | 0.74 | 0.87 | 0.80 | 0.69 | 0.92 | 0.77 | 0.68 | 0.86 | 0.77 | 0.66 | 0.91 | 0.80 | 0.67 | 0.96 |
|  |  |  | Endocrine | 0.92 | 0.86 | 0.99 | 0.98 | 0.86 | 1.13 | 0.84 | 0.76 | 0.93 | 0.90 | 0.78 | 1.03 | 0.95 | 0.81 | 1.12 |
|  |  |  | Neurological | 0.60 | 0.51 | 0.70 | 0.70 | 0.51 | 0.96 | 0.49 | 0.40 | 0.60 | 0.46 | 0.35 | 0.61 | 0.60 | 0.42 | 0.84 |
|  |  |  | Respiratory | 0.99 | 0.92 | 1.06 | 1.13 | 0.99 | 1.29 | 1.03 | 0.93 | 1.15 | 0.84 | 0.74 | 0.95 | 0.89 | 0.78 | 1.01 |

TR = ratio of the survival times between the exposed and reference groups; LB CI and UB CI = Lower and upper bounds of the 95% confidence intervals of the ratio of the survival times

The statistical models presented include: COVID + Disability + COVID-19 x disability + Age + Gender + Race/Ethnicity + Underlying Medical Conditions.

The ‘Other disability’ category was not analyzed separately but individuals with a diagnosis in this category were included in the ‘Any disability’ category. Disability types are not mutually exclusive. Regardless of multiple disability types, all individuals are only counted once in the ‘Any disability’ category.

**Table S5.** **Parametric Accelerated Failure Time (AFT) Models and Unadjusted and Adjusted Ratios of Days to Outcome for those with COVID-19 and Disability Compared to Referents and Stratified by Age**

|  | **Referent** | |  | **<18 years** | | | **18-49 years** | | | **>=50 years** | | |
| --- | --- | --- | --- | --- | --- | --- | --- | --- | --- | --- | --- | --- |
|  | **COVID** | **Disability** | **Outcome** | **TR** | **LB** | **UB** | **TR** | **LB** | **UB** | **TR** | **LB** | **UB** |
| A. | No | Disability | Circulatory | 0.39 | 0.15 | 0.99 | 0.42 | 0.31 | 0.56 | 0.51 | 0.46 | 0.55 |
|  |  |  | Endocrine | 0.42 | 0.26 | 0.68 | 0.50 | 0.41 | 0.61 | 0.52 | 0.48 | 0.56 |
|  |  |  | Neurological | 0.37 | 0.12 | 1.12 | 0.35 | 0.21 | 0.58 | 0.54 | 0.45 | 0.63 |
|  |  |  | Respiratory | 0.38 | 0.31 | 0.46 | 0.33 | 0.28 | 0.39 | 0.39 | 0.36 | 0.43 |
|  |  |  |  |  |  |  |  |  |  |  |  |  |
| B. | No | No disability | Circulatory | 0.38 | 0.15 | 0.96 | 0.30 | 0.22 | 0.39 | 0.37 | 0.34 | 0.40 |
|  |  |  | Endocrine | 0.26 | 0.16 | 0.42 | 0.40 | 0.33 | 0.48 | 0.49 | 0.45 | 0.53 |
|  |  |  | Neurological | 0.17 | 0.06 | 0.51 | 0.25 | 0.16 | 0.39 | 0.29 | 0.24 | 0.34 |
|  |  |  | Respiratory | 0.30 | 0.25 | 0.36 | 0.27 | 0.23 | 0.31 | 0.40 | 0.37 | 0.43 |
|  |  |  |  |  |  |  |  |  |  |  |  |  |
| C. | Yes | No disability | Circulatory | 0.84 | 0.33 | 2.17 | 0.76 | 0.57 | 1.00 | 0.70 | 0.64 | 0.77 |
|  |  |  | Endocrine | 0.67 | 0.40 | 1.10 | 1.00 | 0.83 | 1.22 | 0.91 | 0.84 | 0.99 |
|  |  |  | Neurological | 0.34 | 0.10 | 1.14 | 0.73 | 0.45 | 1.17 | 0.52 | 0.44 | 0.62 |
|  |  |  | Respiratory | 0.91 | 0.75 | 1.11 | 0.89 | 0.76 | 1.04 | 1.03 | 0.95 | 1.13 |

TR = ratio of the survival times between the exposed and reference groups; LB CI and UB CI = Lower and upper bounds of the 95% confidence intervals of the ratio of the survival times

The statistical models presented include: COVID + Disability + COVID-19 x disability + Age + Gender + Race/Ethnicity + Underlying Medical Conditions.

**Table S6.** **Parametric Accelerated Failure Time (AFT) Models and Unadjusted and Adjusted Ratios of Days to Outcome for those with and without COVID-19 and/or Disability**

|  |  |  |  | **Time Ratio** | | | **Estimated Days Earlier to Outcome (Reference 100 days)** | | |
| --- | --- | --- | --- | --- | --- | --- | --- | --- | --- |
|  | Exposed | Reference | Outcome | TR | LB CI | UB CI | Days | LB | UB |
| A. | COVID-19 + Disability | No COVID-19 + Disability | Circulatory | 0.50 | 0.46 | 0.55 | 50 | 45 | 54 |
|  |  |  | Endocrine | 0.52 | 0.48 | 0.57 | 48 | 43 | 52 |
|  |  |  | Neurological | 0.48 | 0.41 | 0.57 | 52 | 43 | 59 |
|  |  |  | Respiratory | 0.41 | 0.38 | 0.44 | 59 | 56 | 62 |
| B. | COVID-19 + Disability | COVID-19 +  No disability | Circulatory | 0.87 | 0.80 | 0.96 | 13 | 20 | 4 |
|  |  |  | Endocrine | 0.98 | 0.90 | 1.06 | 2 | 10 | -6 |
|  |  |  | Neurological | 0.75 | 0.63 | 0.88 | 25 | 37 | 12 |
|  |  |  | Respiratory | 1.09 | 1.01 | 1.18 | -9 | -1 | -18 |
| C. | COVID-19 + Disability | No COVID-19 +  No disability | Circulatory | 0.46 | 0.42 | 0.50 | 54 | 50 | 58 |
|  |  |  | Endocrine | 0.51 | 0.48 | 0.55 | 49 | 45 | 52 |
|  |  |  | Neurological | 0.32 | 0.27 | 0.38 | 68 | 62 | 73 |
|  |  |  | Respiratory | 0.42 | 0.39 | 0.45 | 58 | 55 | 61 |

Note: This analysis corresponds to the sensitivity analysis described in the manuscript and uses an alternative approach to assigning index dates for patient controls.

TR = ratio of the event times between the exposed and reference groups; LB CI and UB CI = Lower and upper bounds of the 95% confidence intervals of the ratio of the survival times

The statistical models presented include: COVID-19 + Disability + COVID-19 x disability + Age + Gender + Race/Ethnicity + Underlying Medical Conditions + Pre-pandemic Healthcare Utilization.

**Table S7. Top Five Diagnoses Reported in Primary Care Visits, Stratified by Disability, COVID Status, and Clinical Outcome Group**

| **Disability Status** | **COVID Status** | **Clinical Outcome** | **Diagnostic Code** | **Count** | **Percentage** | **Clinical Description** |
| --- | --- | --- | --- | --- | --- | --- |
| Disability | COVID | Circulatory | I10 | 129 | 15.2 | Essential (primary) hypertension |
|  |  |  | I2510 | 39 | 4.6 | Athscl heart disease of native coronary artery w/o ang pctrs |
|  |  |  | I959 | 33 | 3.9 | Hypotension, unspecified |
|  |  |  | I129 | 31 | 3.7 | Hypertensive chronic kidney disease w stg 1-4/unsp chr kdny |
|  |  |  | I509 | 31 | 3.7 | Heart failure, unspecified |
|  |  | Endocrine | E782 | 93 | 8.8 | Mixed hyperlipidemia |
|  |  |  | E785 | 80 | 7.6 | Hyperlipidemia, unspecified |
|  |  |  | E559 | 67 | 6.4 | Vitamin D deficiency, unspecified |
|  |  |  | E1165 | 50 | 4.7 | Type 2 diabetes mellitus with hyperglycemia |
|  |  |  | E119 | 49 | 4.6 | Type 2 diabetes mellitus without complications |
|  |  | Neurological | R4182 | 15 | 7.9 | Altered mental status, unspecified |
|  |  |  | R2681 | 14 | 7.3 | Unsteadiness on feet |
|  |  |  | R296 | 13 | 6.8 | Repeated falls |
|  |  |  | G629 | 12 | 6.3 | Polyneuropathy, unspecified |
|  |  |  | F0390 | 10 | 5.2 | Unsp dementia, unsp severity, without beh/psych/mood/anx |
|  |  | Respiratory | J069 | 147 | 12.8 | Acute upper respiratory infection, unspecified |
|  |  |  | J0190 | 67 | 5.8 | Acute sinusitis, unspecified |
|  |  |  | J209 | 65 | 5.7 | Acute bronchitis, unspecified |
|  |  |  | J029 | 63 | 5.5 | Acute pharyngitis, unspecified |
|  |  |  | J189 | 58 | 5.1 | Pneumonia, unspecified organism |
|  | No COVID | Circulatory | I10 | 1628 | 19.2 | Essential (primary) hypertension |
|  |  |  | I2510 | 423 | 5 | Athscl heart disease of native coronary artery w/o ang pctrs |
|  |  |  | I4891 | 341 | 4 | Unspecified atrial fibrillation |
|  |  |  | I129 | 316 | 3.7 | Hypertensive chronic kidney disease w stg 1-4/unsp chr kdny |
|  |  |  | I509 | 294 | 3.5 | Heart failure, unspecified |
|  |  | Endocrine | E782 | 1023 | 9.8 | Mixed hyperlipidemia |
|  |  |  | E785 | 878 | 8.4 | Hyperlipidemia, unspecified |
|  |  |  | E559 | 797 | 7.6 | Vitamin D deficiency, unspecified |
|  |  |  | E119 | 602 | 5.8 | Type 2 diabetes mellitus without complications |
|  |  |  | E039 | 586 | 5.6 | Hypothyroidism, unspecified |
|  |  | Neurological | R296 | 128 | 7.9 | Repeated falls |
|  |  |  | R413 | 118 | 7.3 | Other amnesia |
|  |  |  | F0390 | 105 | 6.5 | Unsp dementia, unsp severity, without beh/psych/mood/anx |
|  |  |  | G629 | 95 | 5.9 | Polyneuropathy, unspecified |
|  |  |  | R2689 | 78 | 4.8 | Other abnormalities of gait and mobility |
|  |  | Respiratory | J069 | 1180 | 13.3 | Acute upper respiratory infection, unspecified |
|  |  |  | J029 | 781 | 8.8 | Acute pharyngitis, unspecified |
|  |  |  | J0190 | 602 | 6.8 | Acute sinusitis, unspecified |
|  |  |  | J309 | 513 | 5.8 | Allergic rhinitis, unspecified |
|  |  |  | J209 | 404 | 4.5 | Acute bronchitis, unspecified |
| No disability | No COVID | Circulatory | I10 | 31050 | 37.7 | Essential (primary) hypertension |
|  |  |  | I2510 | 4860 | 5.9 | Athscl heart disease of native coronary artery w/o ang pctrs |
|  |  |  | I129 | 2863 | 3.5 | Hypertensive chronic kidney disease w stg 1-4/unsp chr kdny |
|  |  |  | I4891 | 2785 | 3.4 | Unspecified atrial fibrillation |
|  |  |  | I480 | 2122 | 2.6 | Paroxysmal atrial fibrillation |
|  |  | Endocrine | E782 | 17102 | 13 | Mixed hyperlipidemia |
|  |  |  | E785 | 14726 | 11.2 | Hyperlipidemia, unspecified |
|  |  |  | E559 | 10526 | 8 | Vitamin D deficiency, unspecified |
|  |  |  | E119 | 9684 | 7.4 | Type 2 diabetes mellitus without complications |
|  |  |  | E039 | 7347 | 5.6 | Hypothyroidism, unspecified |
|  |  | Neurological | G629 | 750 | 8.9 | Polyneuropathy, unspecified |
|  |  |  | R413 | 664 | 7.9 | Other amnesia |
|  |  |  | G43909 | 660 | 7.9 | Migraine, unsp, not intractable, without status migrainosus |
|  |  |  | R296 | 432 | 5.1 | Repeated falls |
|  |  |  | F0390 | 328 | 3.9 | Unsp dementia, unsp severity, without beh/psych/mood/anx |
|  |  | Respiratory | J069 | 22680 | 16.3 | Acute upper respiratory infection, unspecified |
|  |  |  | J029 | 15197 | 11 | Acute pharyngitis, unspecified |
|  |  |  | J0190 | 13227 | 9.5 | Acute sinusitis, unspecified |
|  |  |  | J309 | 7178 | 5.2 | Allergic rhinitis, unspecified |
|  |  |  | J209 | 6348 | 4.6 | Acute bronchitis, unspecified |
|  | COVID | Circulatory | I10 | 2145 | 32 | Essential (primary) hypertension |
|  |  |  | I2510 | 381 | 5.7 | Athscl heart disease of native coronary artery w/o ang pctrs |
|  |  |  | I129 | 250 | 3.7 | Hypertensive chronic kidney disease w stg 1-4/unsp chr kdny |
|  |  |  | I4891 | 229 | 3.4 | Unspecified atrial fibrillation |
|  |  |  | I480 | 201 | 3 | Paroxysmal atrial fibrillation |
|  |  | Endocrine | E782 | 1223 | 11.2 | Mixed hyperlipidemia |
|  |  |  | E785 | 1053 | 9.7 | Hyperlipidemia, unspecified |
|  |  |  | E559 | 841 | 7.7 | Vitamin D deficiency, unspecified |
|  |  |  | E119 | 796 | 7.3 | Type 2 diabetes mellitus without complications |
|  |  |  | E1165 | 634 | 5.8 | Type 2 diabetes mellitus with hyperglycemia |
|  |  | Neurological | R413 | 80 | 9.3 | Other amnesia |
|  |  |  | G43909 | 72 | 8.4 | Migraine, unsp, not intractable, without status migrainosus |
|  |  |  | G629 | 72 | 8.4 | Polyneuropathy, unspecified |
|  |  |  | R296 | 36 | 4.2 | Repeated falls |
|  |  |  | G44209 | 31 | 3.6 | Tension-type headache, unspecified, not intractable |
|  |  | Respiratory | J069 | 1931 | 13.4 | Acute upper respiratory infection, unspecified |
|  |  |  | J029 | 1403 | 9.7 | Acute pharyngitis, unspecified |
|  |  |  | J0190 | 1300 | 9 | Acute sinusitis, unspecified |
|  |  |  | J209 | 749 | 5.2 | Acute bronchitis, unspecified |
|  |  |  | J0100 | 693 | 4.8 | Acute maxillary sinusitis, unspecified |
